# Complement Receptor 1 availability on red blood cell surface modulates *Plasmodium vivax* invasion of human reticulocytes

**DOI:** 10.1038/s41598-019-45228-6

**Published:** 2019-06-20

**Authors:** Surendra Kumar Prajapati, Céline Borlon, Eduard Rovira-Vallbona, Jakub Gruszczyk, Sebastien Menant, Wai-Hong Tham, Johanna Helena Kattenberg, Elizabeth Villasis, Katlijn De Meulenaere, Dionicia Gamboa, Joseph Vinetz, Ricardo Fujita, Xa Nguyen Xuan, Marcelo Urbano Ferreira, Carlos H. Niño, Manuel A. Patarroyo, Gregory Spanakos, Luc Kestens, Jan Van Den Abbeele, Anna Rosanas-Urgell

**Affiliations:** 1 Department of Biomedical Sciences, Institute of Tropical Medicine, Antwerp, Belgium; 2 The Walter and Eliza Hall Institute of Medical Research, Parkville, Victoria, Australia; 3 Department of Medical Biology, The University of Melbourne, Parkville, Victoria, Australia; 4 Departamento de Ciencias Celulares y Moleculares, Universidad Peruana Cayetano Heredia, Lima, Peru; 5 Adrem Data Lab, University of Antwerp, Antwerp, Belgium; 6 Section of Infectious Diseases, Department of Internal Medicine, Yale School of Medicine, New Haven, USA; 7 Centro de Genética y Biología Molecular, Universidad de San Martín de Porres, Lima, Peru; 8 National Institute of Malariology, Parasitology and Entomology, Hanoi, Vietnam; 9 Departamento de Parasitologia, Universidade de São Paulo, São Paulo, Brazil; 10 Molecular Biology and Immunology Department, Fundación Instituto de Inmunología de Colombia (FIDIC), Bogotá, Colombia; 11 Basic Sciences Department, Universidad del Rosario, Bogotá, Colombia; 12 Present Address: Hellenic Centre for Diseases Control and Prevention, Maroussi, Greece; 13 Department of Microbiology and Immunology, Uniformed Services University of Health Sciences, Bethesda, USA

**Keywords:** Genetic linkage study, Malaria

## Abstract

*Plasmodium vivax* parasites preferentially invade reticulocyte cells in a multistep process that is still poorly understood. In this study, we used *ex vivo* invasion assays and population genetic analyses to investigate the involvement of complement receptor 1 (CR1) in *P*. *vivax* invasion. First, we observed that *P*. *vivax* invasion of reticulocytes was consistently reduced when CR1 surface expression was reduced through enzymatic cleavage, in the presence of naturally low-CR1-expressing cells compared with high-CR1-expressing cells, and with the addition of soluble CR1, a known inhibitor of *P*. *falciparum* invasion. Immuno-precipitation experiments with *P*. *vivax* Reticulocyte Binding Proteins showed no evidence of complex formation. In addition, analysis of CR1 genetic data for worldwide human populations with different exposure to malaria parasites show significantly higher frequency of CR1 alleles associated with low receptor expression on the surface of RBCs and higher linkage disequilibrium in human populations exposed to *P*. *vivax* malaria compared with unexposed populations. These results are consistent with a positive selection of low-CR1-expressing alleles in vivax-endemic areas. Collectively, our findings demonstrate that CR1 availability on the surface of RBCs modulates *P*. *vivax* invasion. The identification of new molecular interactions is crucial to guiding the rational development of new therapeutic interventions against vivax malaria.

## Introduction

*Plasmodium vivax* is the most widespread human malaria parasite outside sub-Saharan Africa, with 2.5 billion people at risk of infection and tens of millions of cases every year^1^. During the asexual cycle, parasites in the human blood invade, grow, and multiply inside young red blood cells (RBCs), bursting at the schizont stage and releasing merozoites into the blood circulation to invade new reticulocytes. RBC invasion is a complex, multi-step process that involves merozoite attachment, apical reorientation, tight junction formation, and host cell invagination and penetration. In addition, it requires the orchestration of multiple ligand-receptor interactions throughout the different steps of the process^2^. While many of the parasite-host interactions engaged in invasion have been characterized through well-established culture techniques and advanced genetic technologies for *Plasmodium falciparum*^2,3^, research on *P*. *vivax* invasion mechanisms continues to lag behind owing to the lack of a reliable long-term *in vitro* culture system dependent on a continuous source of reticulocytes^4,5^. A deeper understanding of the mechanisms involved in the process of *P*. *vivax* invasion is essential for designing meaningful strategies to prevent *P*. *vivax* infections. However, the only well-characterized, essential ligand-receptor interaction that has been identified to date is that between the Duffy antigen receptor for chemokines (DARC) on RBCs and *P*. *vivax* Duffy binding protein (PvDBP)^6^. *P*. *vivax* infections are rare in sub-Saharan African populations, where a silencing mutation in the Duffy blood group is present at near fixation levels^7^. However, recent reports of *P*. *vivax* infections in Duffy-negative individuals^8^, together with recently described receptor-ligand interactions^9–11^ and potential parasite ligands to unknown receptors^12^, paint a much more complex scenario with multiple host-parasite interactions yet to be well characterized.

During reticulocyte maturation, the RBC membrane goes through intense remodeling of its surface in a process that results in a significant reduction and loss of receptors such as complement receptor 1 (CR1, CD35). CR1 is a type 1 transmembrane protein whose expression is reduced 3.5-fold during reticulocyte maturation^13^. It has an immune-regulatory role in complement activation and removes C3b- and C4b-containing immune complexes from the blood circulation^14^. It is also a known receptor for *P*. *falciparum* invasion through binding with the PfRh4 parasite ligand^15,16^ and for rosetting through interaction with the parasite’s erythrocyte membrane protein-1 (PfEMP-1)^17^.

CR1 protein levels on the surface of RBCs are genetically determined by low (L) and high (H) expression alleles that result in the production of high (HH), medium (HL), or low (LL) levels of CR1. Two CR1 SNPs (intron 27 [A > T: rs11118133]^18^ and exon 22 [A > G: rs2274567])^16,19^ have been identified in association with low CR1 levels in populations from Europe^19^, America^18,20^, Asia^21^, and Melanesia^19^. Although this association has not been identified in African populations (Malians and African-Americans)^20,22^, the two SNPs are under linkage disequilibrium (LD) in both Caucasians and Africans^23^. An association between low CR1 expression and protection against severe *P*. *falciparum* malaria has been reported in some epidemiological studies^19,24–26^ but not in others^21,27^, illustrating a complex relationship between CR1 and susceptibility to *P*. *falciparum* infection and disease. To the best of our knowledge, the involvement of CR1 in *P*. *vivax* invasion has not yet been investigated.

We hypothesize that CR1 on the surface of reticulocytes is involved in *P*. *vivax* invasion and that polymorphisms in the *CR1* gene associated with low CR1 expression have been positively selected in *P*. *vivax-*exposed populations because of their protective effect, *i*.*e*., their ability to decrease the efficiency of *P*. *vivax* invasion. To explore this hypothesis, we performed *ex vivo* invasion assays under different experimental conditions and investigated signals of positive selection in the *CR1* gene using SNP data from worldwide populations.

## Results

### *P*. *vivax* invasion is reduced in trypsin-treated reticulocyte-enriched red blood cells

**Figure 1:**
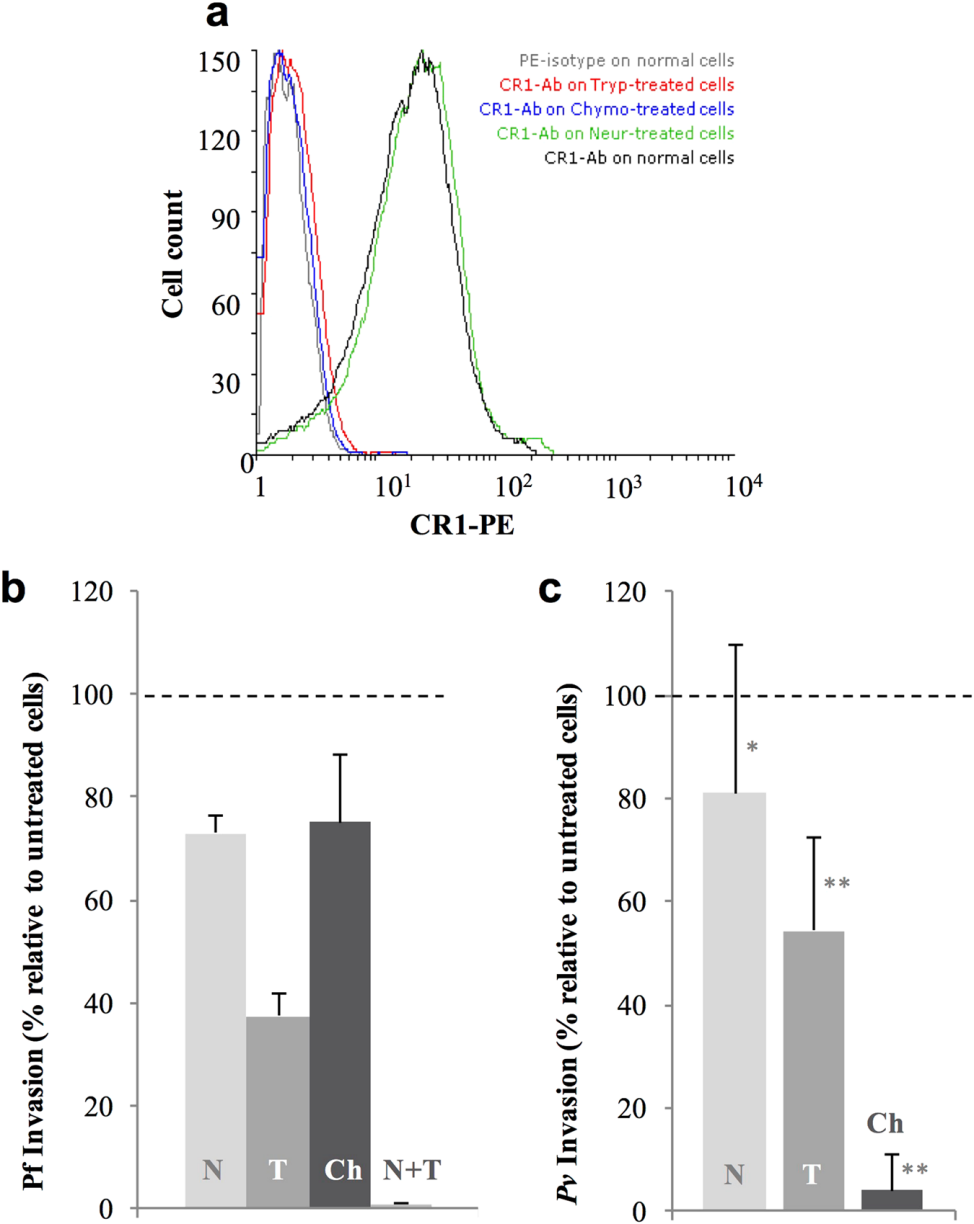
Reduced *P*. *vivax* reticulocyte invasion after enzymatic treatment of reticulocyte-enriched red blood cells (reRBCs). ReRBC samples were treated with neuraminidase (N), trypsin (T), and chymotrypsin (Ch). Experiments were performed using two reRBC samples: HCR32 and HCR43 with CR1 mean florescence intensity (MFI) of 38.47 and 52.73, respectively. (**a**) CR1 cleavage efficiency was assessed by flow cytometry (FACSCalibur 4-color flow cytometer) with anti-CR1 antibody on treated and untreated cells (results shown for HCR43). Invasion assays were performed in duplicate with the 3D7 *P*. *falciparum* strain (**b**) and with five *P*. *vivax* isolates. (**c**) Invasion reduction is relative to untreated cells with standard deviation. *p ≥ 0.13 and **p ≤ 0.043.

Enzymatic cleavage of red blood cell receptors has been extensively used to investigate invasion pathways in *P*. *falciparum* parasites^28–30^. To investigate the profile of *P*. *vivax* invasion, high-CR1-expressing (H-CR1) reticulocyte-enriched RBCs (reRBCs) (mean fluorescence intensity [MFI]: 31.81 and 38.47) (Table 1, Figure S1) were treated with neuraminidase (removes sialic acid residues), trypsin (cleaves several unknown receptors in addition to CR1) and chymotrypsin (cleaves several unknown receptors in addition to CR1 and DARC). Efficient cleavage of CR1 was confirmed by flow cytometry following antibody labeling with anti-CR1 antibody (Fig. 1a). The capacity of the treated cells for invasion was confirmed with the *P*. *falciparum* 3D7 strain (Fig. 1b), which results are in agreement with previous findings^31–33^. *P*. *vivax* invasion was reduced by 18.9% (SD 28.74%, N = 5 isolates, signed rank p = 0.13) with neuraminidase treatment, 45.69% (SD 18.26%, N = 5 isolates, signed rank p = 0.043, range 33–78%) with trypsin treatment, and 96% (SD 7%, N = 5 isolates, signed rank p = 0.043) with chymotrypsin treatment (Fig. 1c, Table S1). The results demonstrate that *P*. *vivax* invasion is sensitive to treatment with chymotrypsin and trypsin. The latest reduced *P*. *vivax* invasion efficiency by half in the presence of an intact DARC, suggesting that one or more trypsin-sensitive receptors (e.g., CR1) are involved in the process of *P*. *vivax* invasion. On the other hand, although *P*. *vivax* invasion was not significantly affected by neuraminidase treatment, a potential interacting role of sialic acid receptors cannot be excluded and deserve further exploration in future assays.

**Table 1:** Description of reticulocyte-enriched red blood cell (reRBC) samples for *ex vivo* invasion assays.

ReRBC sample	Duffy phenotype	Blood group	Reticulocyte %	CR1 exon 22	CR1 (MFI)	CD71 (MFI)	Invasion assays			
							Enzyme treatment	sCR1	Low CR1	High CR1
HCR29	a + b+	O+	40	HH#	42.57	3810		√		
HCR32	a + b+	O+	40	HL#	38.47	ND	√			
HCR40	a + b+	O+	28	HH	52.73	3379		√		√
HCR43	a + b+	O+	36	HH	31.8	3725	√	√		√
HCR47	a + b+	A−	41	HL	39.9	3366		√		√
HCR14	a + b+	A+	25	HL#	19.69	3966			√	
HCR63	a + b−	O+	56	HH	ND	ND		√		
HCR49	a − b+	A−	69	HL	40.86	ND				
HCR48	a + b+	O+	18	HH	29.96	ND				
HCR34	a + b+	A−	70	HL	31.40	ND				
HCR39	a + b+	O+	25	HH	29.25	ND				
HCR17	a + b−	O+	35	HL	24.5	ND				
HCR33	a − b+	O+	42	HH	54,53	ND				

Abbreviations: H: high-CR1 allele, L: low-CR1 allele, sCR1: soluble recombinant CR1 protein, MFI: Mean fluorescence intensity, Low CR1: invasion assays using reRBCs with low CR1 expression phenotype, High CR1: invasion assays using reRBCs with high CR1 expression phenotype. #: CR1 exon 22 genotypes were determined using cDNA. ND: not done. ReRBC samples used in specific invasion assays are indicated (√).

### *P*. *vivax* invasion rate is dependent on CR1 expression levels

**Figure 2:**
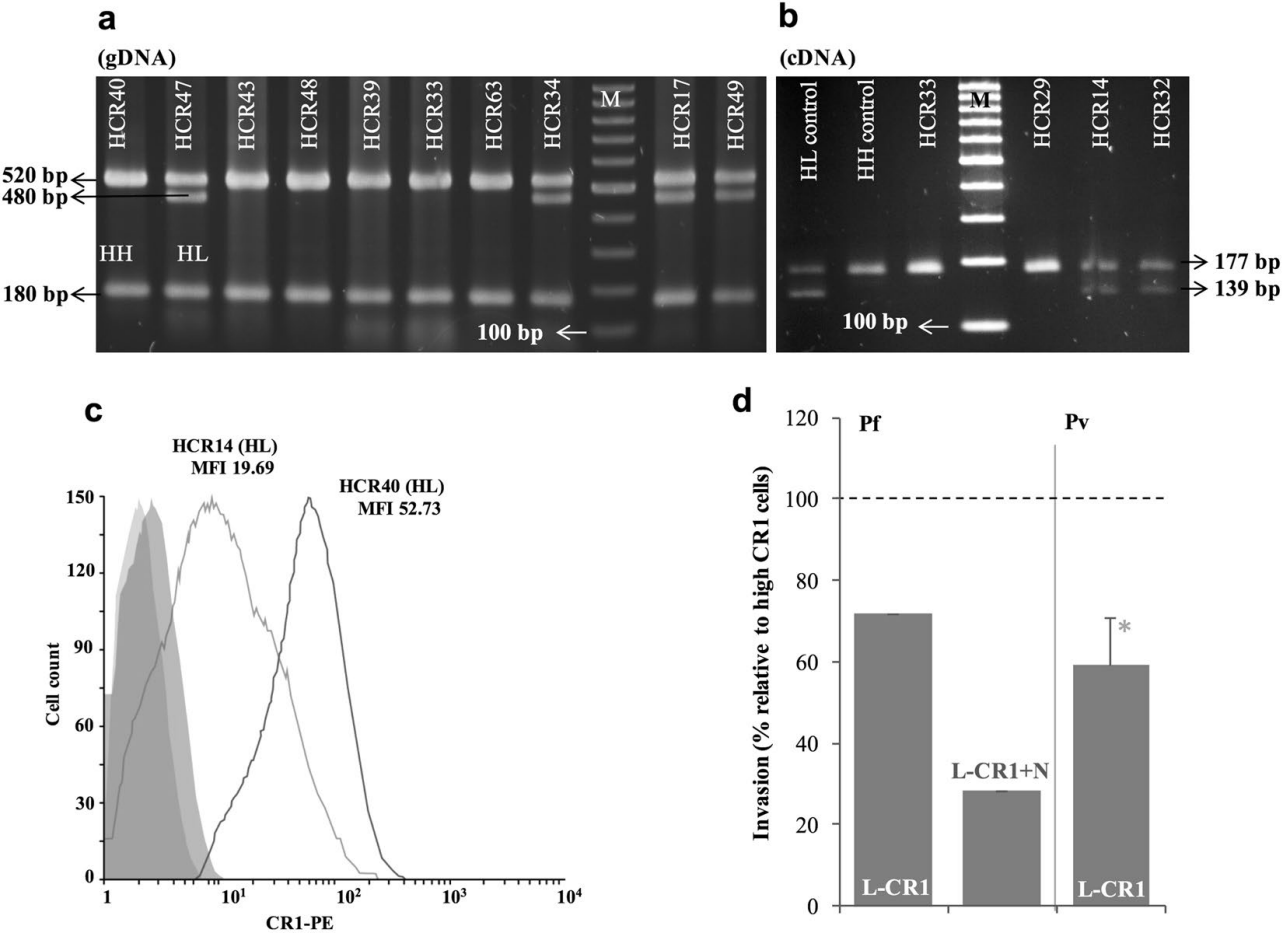
*P*. *vivax* invasion efficiency is dependent on CR1 availability. CR1 characterization in reticulocyte-enriched red blood cell samples. CR1 exon 22 genotype was determined by PCR-RFLP on genomic DNA (**a**) and cDNA (**b**) (panel a and b correspond to two different gels). (**c**) Shows the level of CR1 expression by flow cytometry (FACSCalibur 4-color flow cytometer) as mean fluorescence intensity (MFI) for sample HCR14 and sample HCR40 with the corresponding exon 22 genotypes and these two samples are example to show Low and High CR1 expression differentiation by flow cytometer. (**d**) Invasion assays were first performed with *P*. *falciparum* 3D7 on low-CR1-expressing (L-CR1) cells. Invasion of neuraminidase (N)-treated cells was significantly lower than that of high-CR1-expressing (H-CR1) cells (N = 1). *P*. *vivax* invasion of L-CR1 cells was significantly lower (*p = 0.027) than that of H-CR1 cells (N = 6 isolates). Bars indicate invasion rate relative to H-CR1 cells with SD. HH: homozygote, HL: heterozygote for exon 22 (rs2274567).

Expression of CR1 on the surface of RBCs varies among individuals and this variation is correlated with exon 22 and intron 27 SNPs in non-African populations^19,22^. To test whether CR1 availability on the surface of reticulocytes affects the efficiency of *P*. *vivax* invasion, we characterized the CR1 exon 22 genotype and CR1 expression levels on the surface of reRBC samples (Fig. 2, Table 1). We identified seven homozygote (HH) and six heterozygote (HL) reRBC samples for the exon 22 SNP (rs2274567) (Fig. 2a,b, Table S2), and observed variable levels of CR1 expression (MFI range: 19.69–54.53) (Fig. 2c, Table 1). We did not identify any cases of the LL genotype. To determine if CR1 levels affect *P*. *vivax* invasion we compared the invasion rate between reRBC samples expressing high levels of CR1 (High: n = 3; mean MFI: 41.47 SD 10.55) and the sample expressing the lowest levels (Low: n = 1, MFI = 19.69). The level of CR1 expression on L-CR1 reRBC sample was significantly lower than H-CR1 reRBC samples (p = 0.029), while reticulocyte enrichment levels of the L-CR1 reRBC sample (HCR14: 25%) and the H-CR1 samples (HCR40/HCR43/HCR47: 28%/36%/41%) were not significantly different (p = >0.14). In addition, all reRBC expressed similar levels of CD71 on the surface of reRBCs (HCR14/HCR40/HCR43/HCR47: CD71MFI = 3966/3379/3725/3366) and are Duffy a + b+. Collectively, these results suggest no difference in the expression levels of either Duffy receptor or CD71 between low and high CR1 compared reRBCs samples.

In agreement with previous observations^15,16^, *P*. *falciparum* invasion of neuraminidase-treated and -untreated low-CR1-expressing (L-CR1) reRBCs (MFI: 19.69) was 72% and 28% lower than that of H-CR1 reRBCs (mean MFI: 41.47, SD 10.55) (p < 0.05) (Fig. 2d) demonstrating the validity of our experimental system. *P*. *vivax* invasion of a single L-CR1 reRBCs sample was performed using N = 6 isolates, and was 40.8% (SD 11.2%, signed rank p < 0.027) lower than that of H-CR1 reRBCs samples (Fig. 2d). While these results are consistent with a role for CR1 in mediating *P*. *vivax* invasion, additional L-CR1 samples would be necessary to demonstrate whether the impaired invasion is attributable to CR1 levels.

### Soluble recombinant CR1 protein reduces *P*. *vivax* invasion

**Figure 3:**
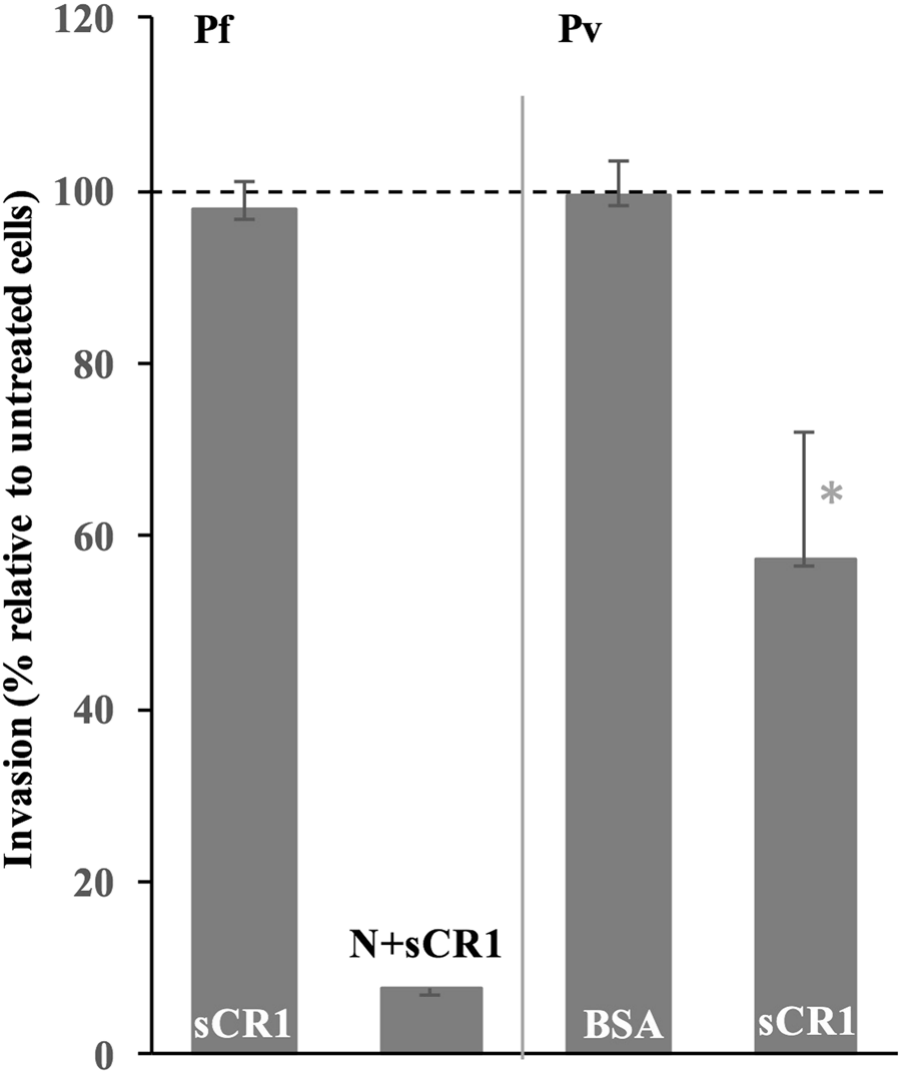
*P*. *vivax* invasion is inhibited by soluble recombinant CR1 (sCR1) protein. *P*. *falciparum* 3D7 invasion of high CR1-expressing neuraminidase (N)-treated cells in the presence of sCR1 was significantly reduced compared with untreated H-CR1 cells (χ^2^ = 55.9, p ≤ 0.001), while *P*. *falciparum* 3D7 invasion efficiency did not vary in the presence of sCR1 on untreated cells (N = 2). *P*. *vivax* invasion of H-CR1 cells in the presence of sCR1 was significantly inhibited relative to H-CR1 without sCR1 (N = 9 isolates). Invasion inhibition was not observed by BSA (N = 5 isolates) or PBS (N = 1 isolate). Invasion efficiency did not significantly vary in the presence of BSA (N = 5 isolates). Bars indicate invasion relative to untreated cells with SD. *p = 0.007.

To further test the role of CR1 in *P*. *vivax* invasion, we performed invasion assays in the presence of soluble recombinant CR1 protein (sCR1). A previous study had demonstrated that in laboratory conditions, incubation of neuraminidase-treated RBCs with sCR1 (50 μg/ml) blocked *P*. *falciparum* invasion^15,16^. In our case, incubation of neuraminidase-treated H-CR1 reRBCs (Table 1) with sCR1 reduced *P*. *falciparum* 3D7 invasion by 90%, confirming previous results (Fig. 3). In the case of *P*. *vivax*, incubation of H-CR1 reRBCs with sCR1 (50 μg/ml) significantly reduced invasion by 42.5% (SD 14.6%, N = 9 isolates, signed rank, p = 0.007) (Fig. 3). These results indicate that sCR1 directly binds to an unrecognized *P*. *vivax* ligand and that this interaction reduces binding between *P*. *vivax* parasites and native CR1 on reticulocytes. The observed reduction in invasion capacity is comparable to that observed for L-CR1 reRBCs compared with H-CR1-reRBCs.

### Lack of binding between sCR1 and recombinant *P*. *vivax* PvRBP parasite ligands

**Figure 4:**
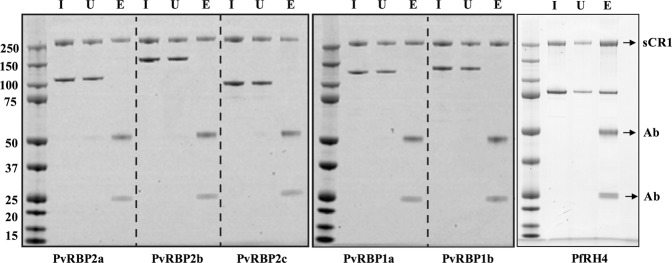
Immuno-precipitation assay to examine PvRBP-soluble CR1 complex formation. An anti-CR1 monoclonal antibody HB8592 was incubated with soluble recombinant CR1 (sCR1) in the presence of either recombinant PfRH4 (right panel) or PvRBP1a and PvRBP1b (middle panel) or PvRBP2a, PvRBP2b and PvRBP2c (left panel) and protein G Sepharose (right, middle and left panels correspond to three different gel blots). Immuno-precipitation eluates were fractionated on SDS-PAGE and visualized using SimplyBlue SafeStain. Molecular weight markers are shown on the left-hand side in kDa for each panel. Soluble CR1 is labeled as sCR1 and the two antibody chains are labeled as Ab. I: Input, U: Unbound and E: Eluate.

PvRBPs are homologues of the PfRh family in *P*. *falciparum*^34^. It is known that PfRh4 binds to CR1 to mediate glycophorin independent parasite invasion^16^. As our previous results indicate that CR1 has a role in *P*. *vivax* invasion, we questioned if a member of the PvRBP family might be the corresponding *P*. *vivax* parasite ligand for CR1. To test this hypothesis, we performed immuno-precipitation experiments using sCR1, PfRH4 (88 kDa) and five recombinant PvRBP fragments, PvRBP1a, PvRBP1b, PvRBP2a, PvRBP2b, and PvRBP2c, which migrate at 118, 133, 114, 152 and 94 kDa respectively under reducing conditions (Fig. 4). While anti-CR1 monoclonal HB8592 successfully immuno-precipitated sCR1, we saw no evidence of complex formation with any of the PvRBP fragments tested, suggesting that PvRBPs are not direct ligands for CR1. On the other hand, as expected, we observed that anti-CR1 monoclonal HB8592 successfully immuno-precipitated sCR1 in complex with PfRh4.

### Biased distribution of CR1 L allele frequency in areas with *P*. *vivax* transmission

**Figure 5:**
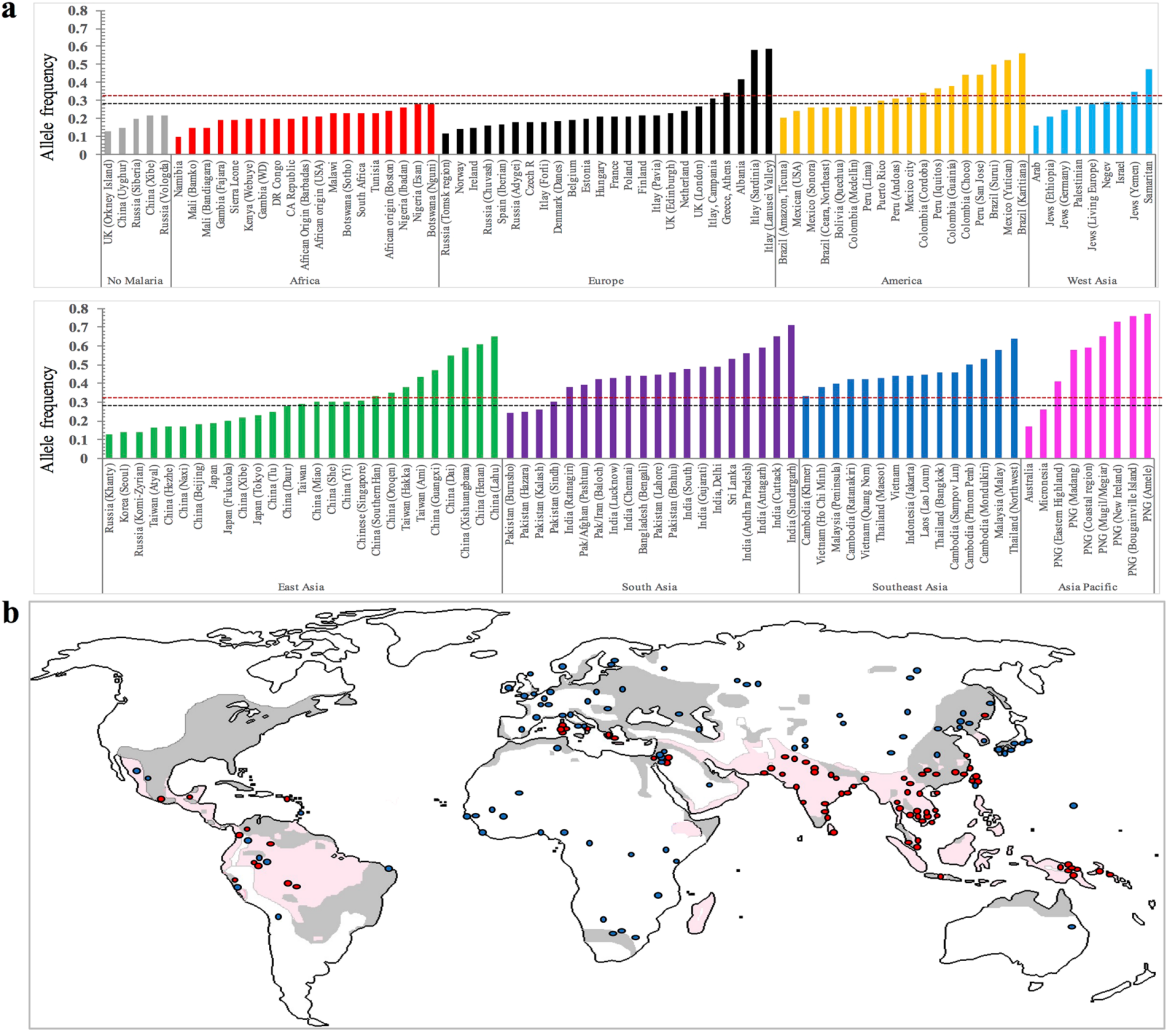
Frequency distribution of low CR1 expression (*L*) allele in global populations. (**a**) Frequency distribution of *L* alleles rs2274567, rs11118131 and rs11118133 in global populations. *L* allele data was obtained for 34,625 samples from 177 sites in 61 countries on five continents (America, Europe, Asia, Africa, and Australia). Populations were categorized into nine groups: eight groups based on the geographical origin of the populations and one based on malaria-free areas. The *L* allele frequency of each population was compared with the mean *L* allele frequency of population groups with no transmission of vivax: (i) the malaria-free group (mean *L* allele = 0.17, range 0.13–0.22) (bars in grey), and (ii) the African group (mean *L* allele = 0.21, range 0.1–0.28) (bars in red). The discontinuous black line represents a significant increase in frequency (*L* ≥ 0.29, p ≤ 0.048) compared with the malaria free group, whereas the discontinuous red line indicates a significant increase in frequency (*L* ≥ 0.33, p ≤ 0.040) compared with the African group. (**b**) The map shows a significant increase in the frequency of the L allele in global populations where *P*. *vivax* transmission is or was stable. Gray shaded areas indicate *P*. *vivax* transmission boundaries between the mid-17^th^ century and the early 20^th^ century and light pink areas indicate transmission boundaries in the late 20^th^ century. *P*. *vivax* approximate boundaries were derived from a previously published figure with permission^79^. Red dots show populations with a significant increase in *L* allele frequency compared with malaria-free areas (*L* ≥ 0.29, p < 0.05), whereas blue dots are indicative of a non-significant increase. The sources of the *L* allele frequencies are described in S3 Table. PNG: Papua New Guinea.

We observed that reduced CR1 availability on reRBCs decreased the efficiency of *P*. *vivax* invasion, even in the presence of an intact DARC. We therefore hypothesized that CR1 genotypes associated with reduced CR1 expression on the surface of reticulocytes may confer protection against *P*. *vivax* infection, possibly reflecting positive selection on *L* alleles in areas exposed to or with a history of exposure to vivax malaria. Positive selection on one allele can be detected by an unusually fast increase in allele frequency in one population relative to another^35^ and extended LD surrounding the allele of interest^36^. To explore our hypothesis, we investigated the frequency of CR1 alleles associated with the L-CR1 phenotype through a meta-analysis of peer-reviewed articles and publicly available data. We first confirmed a LD R^2^ ≥ 0.8 for the CR1 exon 22 (rs2274567) and intron 27 (rs11118133) alleles in populations sampled within the 1000 Genomes Project (Table S2)^23^. Using data mining of publicly available databases we identified a SNP in intron 26 (rs11118131), which was in LD (R^2^ ≥ 0.8) with exon 22 in non-African populations (Table 2). LD between exon 22 and intron 26 was further confirmed by genotyping samples from Vietnam, Thailand, Greece, Belgium, Peru, and Brazil. We included intron 26 in the analysis of CR1 allele frequencies to target additional genotyping data from populations in which exon 22 and intron 27 data were not available. We then analyzed the worldwide distribution of CR1 alleles for intron 27 (rs11118133), exon 22 (rs2274567), and intron 26 (rs11118131), hereafter referred to as *L-CR1* alleles. In total, data mining and in-house genotyping retrieved *L-CR1* allele data for 34,625 individuals from 177 locations across 61 countries (Fig. 5a, Table S3).

**Table 2:** Linkage disequilibrium between CR1 exon 22 (rs2274567) and intron 26 (rs11118131) in global populations.

Geographical region	Country	Sample size	D′	R2
America	Mexico (MXL)	90	1.0	1.0
	Puerto Rico (PUR)	104	0.977	0.9546
	Medellin, Colombia (CLM)	94	1.0	0.9463
	Lima, Peru (PEL)	85	1.0	0.9704
	Andoas, Peru (N = 25) *	25	1.0	1.0
	Iquitos, Peru (N = 23) *	23	1.0	1.0
	Acre, Brazil (N = 24) *	24	1.0	1.0
Europe	Central Europe (CEU)	99	1.0	1.0
	Toscany, Italy (TSI)	107	1.0	1.0
	Finland (FIN)	99	1.0	0.971
	Britain (GBR)	91	1.0	1.0
	Spain (Iberian Pop) (IBS)	107	1.0	0.9668
	Antwerp, Belgium (N = 24) *	24	1.0	1.0
	Greece (N = 32) *	32	1.0	1.0
Africa	African Ancestry (ASW)	61	1.0	0.555
	Ibadan, Nigeria (YRI)	108	0.9715	0.7683
	Isan, Nigeria (ESN)	99	0.9638	0.5883
	Webuye, Kenya (LWK)	99	0.9175	0.6329
	Gambia (MAG)	113	0.9085	0.4766
	Mende, Sierra Leone (MSL)	85	0.9365	0.5366
	African Caribbean (ACB)	96	0.9576	0.6254
South Asia	Lahore, Pakistan (PJL)	96	1.0	0.9791
	Gujrat, India (GIH)	103	1.0	1.0
	Andhra Pradesh, India (ITU)	102	1.0	1.0
	Sri Lanka (STU)	102	1.0	0.9614
	Bangladesh (BEB)	86	1.0	1.0
East Asia	Mae Sot, Thailand (N = 27) *	27	1.0	0.9274
	Quang Nom, Vietnam (N = 24) *	24	0.914	0.836
	Ho Chi Minh, Vietnam (KHV)	99	1.0	0.9788
	Beijing, China (Han) (CHB)	103	1.0	1.0
	Southern Han Chinese (CHS)	105	1.0	1.0
	Xishuandbanna, China (CDX)	93	1.0	1.0
	Japan (JPT)	104	1.0	0.9698

SNP data was retrieved from the 1000 Genomes Project database or in-house genotyping (*). Linkage disequilibrium was analyzed using the LDlink software tool and with CubeX software tool for in-house genotyping data.

We first compared mean *L-CR1* allele frequency in malaria-free areas (defined as areas with no documented history of malaria^37,38^), namely, Orkney Island, UK; Xinjiang province, China; and Siberia and Vologda, Russia (*L-CR1* = 0.17, range 0.13–0.22), with mean frequencies reported for the other populations in the analysis (Table S3). We observed a significantly increased frequency of *L-CR1* in areas with *P*. *vivax* exposure (*L-CR1* ≥ 0.29, p < 0.048) but not in sub-Saharan populations (Fig. 5b), where there is no stable transmission of *P*. *vivax* parasites and *P*. *falciparum* is the only parasite responsible for overall transmission of malaria disease ( > 99.5%). Further, this significant increase in *L-CR1* frequencies (Figure S2) overlapped with the reported transmission intensities of *P*. *vivax* in global populations^39^. A significant increase in *L-CR1* allele frequencies was still observed when we compared the mean frequency for African populations (Fig. 5a,b and Figure S2) (mean *L-CR1* = 0.21, range 0.1–0.28) with that of populations exposed to *P*. *vivax* (alone or with *P*. *falciparum*) (*L-CR1* ≥ 0.33, p < 0.040). These results indicate positive selection on *L-CR1* alleles in vivax-endemic areas.

### LD decay at CR1 locus

**Figure 6:**
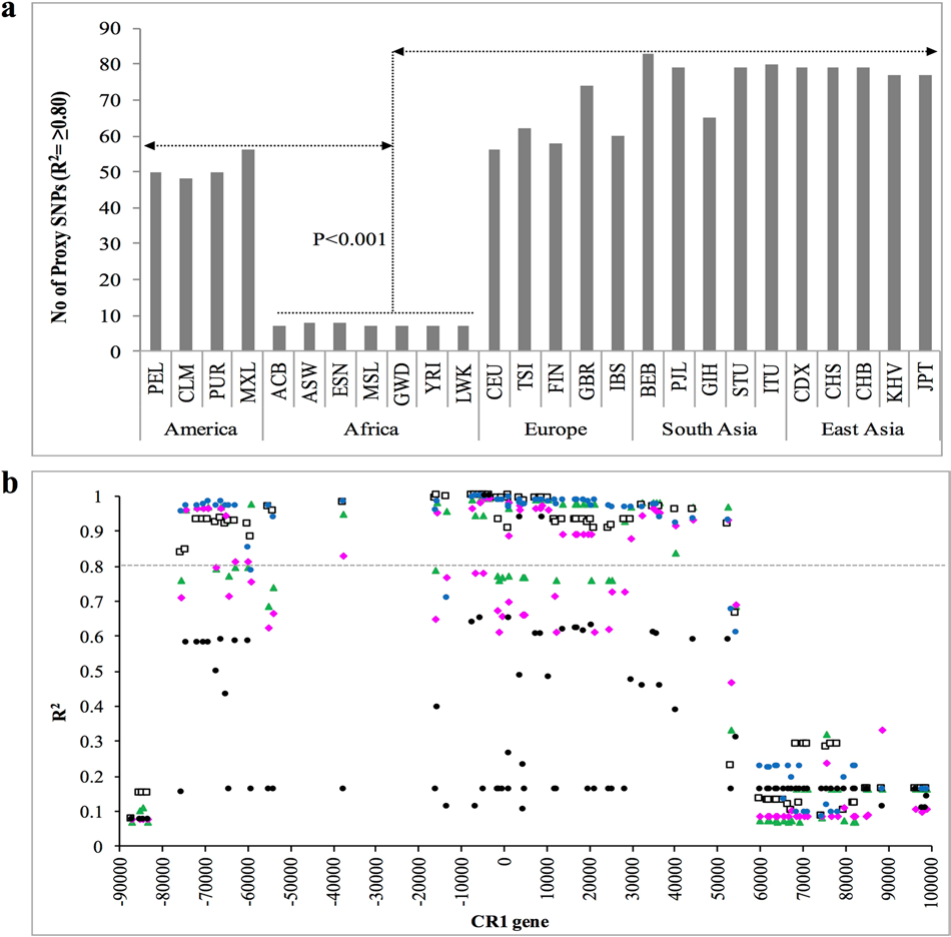
Linkage disequilibrium (LD) analysis suggests a signature of positive selection on the CR1 L allele. (**a**) Number of proxy SNPs in LD with exon 22 in global populations. A 200-Kb region on chromosome 1 spanning the CR1 gene **(**1000 Genomes Project Phase 3 data) was scanned using the LDlink tool to estimate the number of proxy SNPs for exon 22 (rs2274567). A higher number of proxy SNPs for a given SNP is indicative of stronger LD. The number of proxy SNPs for exon 22 was significantly lower in African than non-African populations (1000 Genomes Project Phase 3). (**b**) Physical distance under LD with exon 22 SNP (rs2274567) was analyzed using global population data from the 1000 Genomes Project. Proxy SNPs present in all selected populations were plotted to show the physical distance of each SNP and its R^2^ with respect to exon 22 (rs2274567) (a maximum of one SNPs every 500 bp for better visualization of the LD effect). Data from the 1000 Genomes Project were analyzed by pooling human populations from Africa (ASW, YRI, ESN, LWK, MAG, MSL, ACB), Europe (CEU, TSI, FIN, GBR, IBS), East Asia (JPT, KHV, CHB, CHS, CDX), South Asia (PJL, GIH, ITU, STU, BEB) and America (MXL, PUR, CLM, PEL). Position zero on the X axis indicates the location of exon 22 SNP (rs2274567), while CR1 gene spanned from −85000 to 75000 bp. The Y axis shows R^2^ values. Different shapes indicate SNPs R^2^ values in populations from Europe (green triangle), East Asia (white square), South Asia (blue circle), Africa (black circle) and America (pink rhombus). The dotted black line crossing the Y axis indicates the LD threshold (R^2^ ≥0.8). The physical distance of the CR1 gene under LD in African pooled-populations (dotted line: ~18 Kb; −4,400 bp to 13,500 bp) was significantly lower (p ≤ 0.001) compared to pooled populations from Europe, East Asia, South Asia and America (continuous black line: ~130 kb, −75,000 bp to 54,000 bp).

Positive selection on CR1 exon 22 was further assessed through LD analysis using data from the 1000 Genomes Project Phase 3. We estimated the number of proxy SNPs (SNPs under LD with exon 22) and the physical distance under LD, which indicates non-random association of alleles at multiple loci. We observed a significantly higher number of SNPs with R^2^ ≥ 0.8 in populations exposed to *P*. *vivax* malaria, i.e., populations in East Asia (n = 65–83), South Asia (n = 77–79), Europe (n = 56–74), and South and Central America (n = 48–56), compared with African populations not exposed to *P*. *vivax* malaria (n = 7–8, p < 0.001) (Fig. 6a). In addition, LD decay (resulting from genetic recombination) around the exon 22 SNP was significantly faster in populations from Africa (~18 kb, −4,400 bp to 13,500 bp) than in those from vivax-exposed areas of America, Europe, and Asia (~130 kb, −75,000 bp to 54,000 bp) (p ≤ 0.001) (Fig. 6b). Controls for population stratification is shown in Figure S3 and supplementary information.

## Discussion

*P*. *vivax* reticulocyte invasion is an essential process characterized by a cascade of events involving specific host-parasite interactions that are poorly understood. Our results, from *ex vivo* invasion assays and population genetic analyses, indicate that CR1 availability on the reticulocyte surface modulates *P*. *vivax* invasion even in the presence of intact DARC.

The difficulties associated with investigating *P*. *vivax* invasion mechanisms due to the inability to continuously culture the parasites *in vitro* and limitations of *ex vivo* invasion assays have been extensively discussed elsewhere^4,40^. In brief, several limitations have to be taken into account when performing *P*. *vivax* invasion assays: (1) the low parasite densities (0.5%–0.01%) in most field/clinical isolates of *P*. *vivax*, while >0.1% parasite density is needed to ensure a successful invasion, (2) high proportion of rings (≥80%) are necessary for a successful invasion, (3) *ex vivo* adaptation is extremely variable between parasite isolates and this may be partially explained by inherent characteristics of the parasite^41^, (4) several samples produce a high proportion of gametocyte during the maturation process and are thus discarded, and (5) cryopreserved parasites show lower invasion efficiency than fresh isolates^42^. The limitations in the number of *P*. *vivax* isolates with the correct criteria to perform *ex-vivo* invasion assays and the number of samples that maturate successfully and present an optimal invasion rate (untreated control well), together with the need to reduce assay variability greatly limit the number of conditions that can be tested with one parasite isolate. Nevertheless, the recent increase in the number of publications using *P*. *vivax ex vivo* assays^43,44^ demonstrates the usefulness of this tool to investigate molecular mechanisms related to parasite invasion.

In our experiments, we used cryopreserved *P*. *vivax* isolates and reticulocyte-enriched samples from hemochromatosis donors. In this regard, CD71 availability on reRBCs was confirmed by flow cytometry and by demonstrating the ability of *P*. *vivax* isolates to invade heterogeneous CD71^+ve^ populations (with variable levels of CD71)^44^. In addition, the invasion rates observed are comparable to rates previously reported for different reticulocyte sources^5,40,42,45–47^. Finally, we observed consistently significant invasion inhibition in different experiments using both natural CR1 expression variations of the reRBCs and biochemical approaches to modify CR1 presence at the reRBC surface. In this regard, our findings of a decrease in *P*. *vivax* invasion in trypsin-treated cells contradicts previous observations^48,49^, although a trypsin-sensitive *in vitro* binding between TfR1-PvRBP2b has been demonstrated to be critical for the recognition of reticulocytes during *P*. *vivax* invasion^43^. Discrepancies with previous studies can be at least partially explained by differences in the experimental conditions. In Barnwell *et al*.^48^ the source and enriched proportion of used reticulocytes is unknown, the authors use a single *P*. *vivax* Belem strain maintained in squirrel monkey, invasion is monitored after 8–10 hours of incubation, while information on invasion rates is lacking. In comparison with Malleret *et al*., who used 0.5 mg of trypsin for the digestion treatment^49^, here we used a higher concentration that proved (by flow cytometer) to remove CR1 from the surface of reticulocytes.

Overall, although demonstration of invasion inhibition by anti-CR1 antibody may have strengthened our findings further, our results provide strong support for the involvement of CR1 in the process of *P*. *vivax* invasion.

This role is further supported by our observation of a significant increase in the frequency of *L* alleles and strong LD around the exon 22 SNP in populations where *P*. *vivax* has historically been the major malaria species compared with populations in areas without vivax exposure, such as the African continent^39^. *CR1* alleles associated with a low CR1-expression phenotype may exert a beneficial effect in populations exposed to *P*. *vivax* by reducing the efficiency of invasion, and thus decreasing the risk of infection and disease. Allele frequencies and LD measures correlated well for populations with recent exposure to the *P*. *vivax* parasite (such as Mediterranean, Asian, American, and Pacific populations), while the effect on allele frequencies is faster diluted in populations with past exposure and migration movements (non-Mediterranean European population)^50^.

Population differentiation and extended LD in the *CR1* genomic region has already been reported for the Sardinian population, which had a long history of endemic malaria until shortly after World War II, indicating positive natural selection of CR1 in this population^51^. The authors proposed *P*. *falciparum* as the selective force on the CR1 locus. Indeed, many studies have reported an important role for CR1 in malaria infection and pathogenesis^52^, although investigation of the association between CR1 and *P*. *falciparum* susceptibility has depicted a complex scenario with discrepant results^17,19,21,27,52^. Contradictory results may be explained by multiple factors, including differences in the genetic background of populations and pleiotropic effects of CR1 on different diseases^53–57^, the promiscuous use of the CR1 receptor by *P*. *falciparum* parasites during invasion^15,16^ and rosetting^17^, the complexity of factors influencing CR1 levels in African populations^58^, and (as results from this study indicate) selective pressure exerted by *P*. *vivax* malaria.

Other studies that have previously demonstrated a role for *P*. *vivax* infections in shaping the human genome include studies of Duffy determinants in African individuals and South East Asian ovalocytosis in Papua New Guinea^7,59^.

In addition to trypsin-sensitive *P*. *vivax* invasion, we observed a modest (albeit non-significant) reduction in invasion efficiency in neuraminidase-treated cells. A potential role of sialylated glycoproteins in *P*. *vivax* reticulocyte invasion has not been investigated but deserves further attention.

Finally, we were unable to demonstrate a direct interaction between CR1 and five members of PvRBPs which were thought to play essential roles during reticulocyte invasion^12,60,61^. Further, immunoprecipitation experiments with putative invasion ligands (such as MSP-1^62^, PvRON-1^63^, PvRON-2^64^, PvRON-4^65^ and PvRON-5^66^) may allow to identify invasion ligand used by *P*. *vivax* CR1 invasion pathway.

## Conclusions

We have shown for the first time that CR1 availability on the surface of reticulocytes modulates *P*. *vivax* invasion. We have presented evidence of a recent positive selection of *L* allele expression as a beneficial trait in populations exposed to *P*. *vivax* parasites. Future studies should aim to identify the parasite ligand to CR1 and the possible role of this interaction in Duffy-negative reticulocyte invasion. The identification of new molecular interactions and alternative invasion pathways is crucial for guiding the rational development of therapeutic interventions, i.e., vaccines and drugs, targeting the reduction and prevention of reticulocyte invasion.

## Materials and Methods

### Ethics statement

Ethical approval for the collection of *P*. *vivax* isolates from infected patients in Thailand and Peru was obtained from the Institutional Ethics Committees of the Centre for Clinical Vaccinology and Tropical Medicine, University of Oxford, United Kingdom (OXTREC 027–025), the Faculty of Tropical Medicine, Mahidol University, Bangkok, Thailand, (MUTM 2008-215), and Universidad Peruana Cayetano Heredia, Lima, Peru. Ethical approval for the use of *P*. *vivax* isolates and collection of blood samples from hemochromatosis patients was obtained from the Ethics Committee of the Institute of Tropical Medicine (ITM), Antwerp, Belgium (946/14). Ethical approval to perform CR1 genotyping was obtained from the respective local institutional review boards where samples were collected and analyzed. Informed consent was taken from all the subjects. Human samples from Greece were collected for diagnostic purposes and CR1 allele frequency data were obtained without disclosure of personal details (hence, ethical approval was not required).

### *P. vivax* isolates

*P*. *vivax* isolates were collected from patients with acute *P*. *vivax* infection attending clinics in the Shoklo Malaria Research Unit (SMRU) in Mae Sot, Thailand and from communities close to Iquitos city in the region of Loreto, Peru. A 5-ml sample of blood was collected by venipuncture in lithium-heparinized tubes from patients with *P*. *vivax* malaria mono-infection with a parasite density >1/1000 red blood cells. Samples with ≥80% parasites at the ring stage were platelet- and leukocyte-depleted using a CF11 column as previously described^67^. Purified parasites were frozen in glycerolyte^42^ and stored in liquid nitrogen. The samples were then shipped to ITM, Belgium where the experiments were performed. Table S1 shows a summary of the *P*. *vivax* isolates used in each experiment and the parasite density at the time of collection.

### Reticulocyte-enriched red blood cells from hemochromatosis patients

Reticulocytes were enriched from 450 ml of peripheral blood collected from 13 hemochromatosis patients undergoing therapeutic phlebotomy at the ZNA Sint-Erasmus Hospital in Antwerp, Belgium. Blood samples were collected in SEPACELL bags (Fresenius Kabi) and processed within 48 hours following previously described protocols^68^. In brief, we pass blood through the leucocyte depleting filter (Fresenius Kabi) connected with SEPACELL blood bag. The Duffy phenotype (Fy) was determined by standard serologic methods (DiaMed-ID Micro Typing Systems, DiaMed) and blood grouping was done using a standard ABO antisera kit (Diamed). After depleting platelets and leukocytes, reticulocytes were concentrated by centrifugation (15 min at 400 g without applying a brake) through 70% Percoll^42^ with minor modifications to Percoll concentration, which was adjusted in each sample to obtain a higher reticulocyte yield (Table 1). The proportion of reticulocytes was calculated from the thin smear stained with New Methylene Blue (Sigma) under light microscopy. Samples with a proportion of reticulocytes higher than 25% were used for *P*. *vivax* invasion tests. Reticulocyte freezing and thawing was performed as previously described^42^.

### Invasion inhibition assays

*P*. *vivax* maturation and invasion assays were performed as previously described^42,47^. Briefly, thawed *P*. *vivax* parasites were cultured to the schizont stage. Mature schizonts were concentrated after centrifugation (15 minutes at 1200 g) through 45% Percoll solution, which yields 90–98% enrichment of schizonts. The concentrated mature schizonts were mixed with the reRBC samples in a 1:6 (1 µl schizont and 6 µl reRBC) ratio, and cultivated in McCoy medium (Invitrogen) supplemented with 25% human serum and 0.5% glucose for 25 h. Invasion inhibition assays were set up in a 96-well culture plate with a final volume of 150 µl per well. Parasite cultures were monitored by microscopic examination of the Giemsa-stained thin film.

Invasion rate was defined as the percentage of *P*. *vivax* ring-stage invaded RBCs per 9,000 RBCs 24 hours post-invasion. Invasion inhibition was measured in paired reRBC samples (treated *vs*. untreated control well) using the same parasite isolate. An invasion assay was considered valid when parasitemia of the untreated control well was ≥0.5% with one exception (0.42%). Erythrocyte invasion with 3D7 *P*. *falciparum* parasites were used as a positive control for reRBC invasion.

The used sCR1 is commercially available (Cat No 5748-CD-050, R&D SYSTEMS, USA) and supplied as lyophilized power in sterile condition without a hazard preservative and constituted in sterile PBS (400 µg/ml) as per manufacturer’s instruction. We performed invasion inhibition with sCR1 (50 µg/ml concentration) as described in previous *P*. *falciparum* experiments^15,16^. Bovine serum albumin (BSA: 50 µg/ml) and PBS (12.5%) controls were used to assess the specificity of the assay. Non-hazardous effect of the non-dialyzed sCR1 protein was tested in *P*. *falciparum* 3D7 invasion assays compared to the dialyzed sCR1 protein. Paired comparisons of invasion rates between treatments were analyzed using the non-parametric Wilcoxon signed rank test in STATA v13.

### Enzymatic treatment of red blood cells

Enzymatic treatment of reticulocyte-enriched samples was performed by incubating 10 µl of the cell suspension with 1 mg/ml trypsin (from bovine pancreas, Sigma), 1 mg/ml chymotrypsin (from bovine pancreas, Sigma) and 0.5 U/ml neuraminidase (from *Vibrio cholerae*, Sigma) at 37 °C for 1 hour^69^. CR1 enzymatic cleavage efficiency was tested by FACSCalibur 4-color flow cytometer (BD Biosciences)^15^ and neuraminidase treatment was assessed by performing an agglutination test using lectin from the peanut *Arachis hypogaea*, to detect T-antigen which is exposed after sialic acid cleavage^69^.

### Flow cytometry

Expression levels of CR1 and CD71 were quantified with a FACSCalibur 4-color flow cytometer. Briefly, 200,000 cells were incubated with 5 µl of monoclonal mouse anti-human CR1-PE [E11 mAb] (Biolegend), 20 µl of monoclonal mouse anti-human CD71-APC (BD Biosciences), and 20 µl of monoclonal mouse anti-human DARC/Duffy antigen-FITC (LSBio) for one hour at room temperature. IgG1-PE, IgG1-APC and IgG-FITC conjugated antibodies were used as negative controls. Protein levels are expressed as MFI (Figure S1).

### Protein expression and purification

DNA sequences encoding PvRBP1a (amino acids 160–1170), PvRBP1b (amino acids 140–1275), PvRBP2a (amino acids 160–1135), PvRBP2b (amino acids 161–1454), and PvRBP2c (amino acid 501–1300) were codon-optimized from the Sal-I protein sequences for *Escherichia coli* expression and inserted into a pPROEX HTB expression vector (Life Technologies). Protein expression and purification was performed as described earlier^70^.

### Immuno-precipitation assays

sCR1, recombinant PvRBPs and PfRH4 (88 kDa)^71^ were incubated at 0.05 mg/ml in a reaction volume of 50–100 µl for 1 hour at room temperature. Anti-CR1 monoclonal antibody HB8592 (ATCC) was added to the mixture at 0.02 mg/ml for 1 hour, followed by additional incubation with 10 µl of packed Protein G Sepharose beads to capture the anti-CR1 mAb. The beads were washed three times with 200 µl PBS and the proteins were eluted with equivalent volumes of 2X reducing sample buffer and boiled for three minutes before separating on SDS/PAGE gels. Immuno-precipitation eluates were fractionated on SDS-PAGE and visualized using SimplyBlue SafeStain (Life Technologies), according to the manufacturer’s protocol.

### CR1 SNP genotyping

Genotyping of 582 blood samples (Supplemental data) was performed following previously described protocols^23^. Primer sequences, amplification conditions, and restriction enzymes are detailed in Table S4. For some of the reRBC samples an aliquot for DNA extraction was not available and therefore, we extracted RNA and genotyping PCR was performed on cDNA.

### Allele frequency distribution and linkage disequilibrium (LD) analysis

LD was assessed using data from 1000 Genomes Project Phase 3, genotyping data retrieved from published studies^23,51,57,72^) and in-house genotyped. R^2^ and D’ were measured using LDlink web tool (http://analysistools.nci.nih.gov/LDlink/)^73^, Ensembl database (http://www.ensembl.org/index.html) for biallelic SNPs^74^ and CubeX (http://www.oege.org/software/cubex/)^75^ for genotype data. R^2^ and D’ values ≥0.8 were considered indicative of significant correlation and LD between both SNPs.

CR1 SNP data was extracted from publicly available databases, i.e., the 1000 Genomes Project Phase 3^76^, ALFRED (Allele Frequency Database, U.S. National Sciences Foundation)^77^, and SGVP (Singapore Genome Variation Project)^78^, and from 39 international peer-reviewed publications (Table S3). Comparisons were performed using chi-squared tests. P values < 0.05 were considered significant.

## Electronic supplementary material

Supplementary Information
