# Supplementary material for: Complement Receptor 1 availability on red blood cell surface modulates Plasmodium vivax invasion of human reticulocytes
Source: Sci Rep. 2019 Jun 20;9:8943. doi: 10.1038/s41598-019-45228-6 (PMC6586822; doi:10.1038/s41598-019-45228-6)
Supplement: Supplementary file 1 — Supplementary Information [file 41598_2019_45228_MOESM1_ESM.doc]

**Supplementary information**

**Complement Receptor 1 availability on red blood cell surface modulates *Plasmodium vivax* invasion of human reticulocytes**

**Authors:**

Surendra Kumar Prajapati^1#^, Céline Borlon^1^, Eduard Rovira-Vallbona^1^, Jakub Gruszczyk^2^, Sebastien Menant^2^, Wai-Hong Tham^2,3^, Elizabeth Villasis^4^, Katlijn De Meulenaere^1,5^, Dionicia Gamboa^4^, Joseph Vinetz^4,6^, Ricardo Fujita^7^, Xa Nguyen Xuan^8^, Marcelo Urbano Ferreira^9^, Carlos H. Niño^10^, Manuel A. Patarroyo^10,11^, Gregory Spanakos^12^, Luc Kestens^1^, Jan Van Den Abbeele^1^, Anna Rosanas-Urgell^1^*.

**Materials and methods**

## CR1 SNP genotyping

CR1 genotyping was performed on 582 blood samples collected in the context of other studies in Brazil (Acre, n=100), Peru (Iquitos, Andoas and San Jose, n=160), Colombia (Choco and Cordoba, n=54), Belgium (n=50), Greece (n=83), Albania (n=12), Vietnam (Quang Nam province, n=96), and Thailand (n=27).


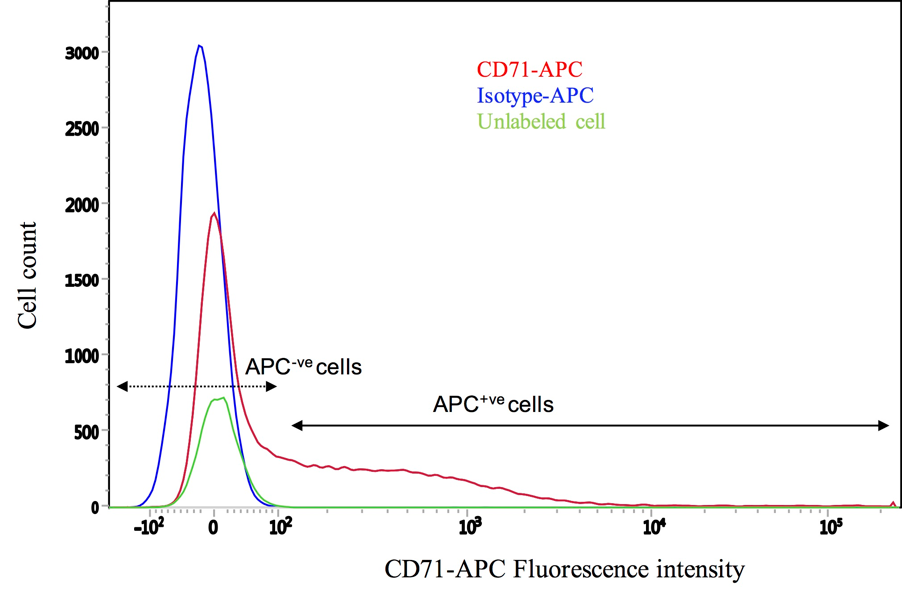


**Figure S1: Quantification of CD71 expression on the surface of reticulocyte cells measured by flow cytometry.**

Expression levels of CD71 on reticulocytes are highest on immature reticulocytes, lower on mature reticulocytes and absent on erythrocytes ^1^. Using FACSVerse 8-color digital flow cytometer, we measured CD71 levels in reticulocyte enriched red blood cell samples from hemochromatosis patient blood by labelling CD71 receptor with CD71-APC antibody (red histogram). IgG-APC positive control and unlabelled cells are shown in blue and green, respectively. The Y axis indicates cell counts and X axis indicates the CD71-APC florescence intensity. The figure shows that enriched reticulocyte samples contain reticulocyte cells at different stage of maturation and thus with varying CD71 levels.


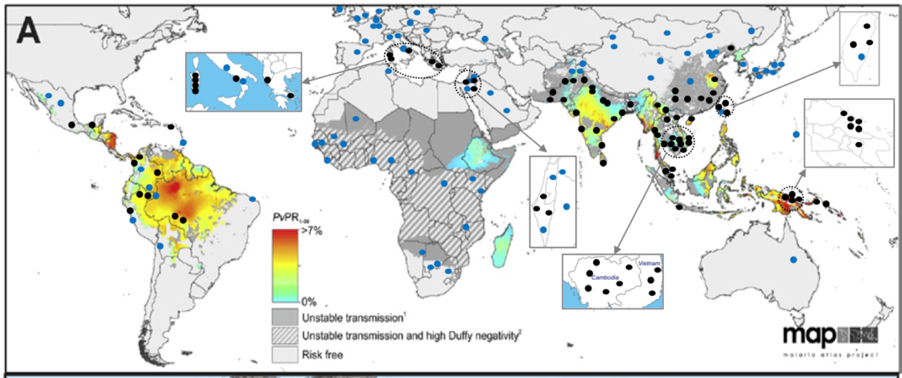


**Figure S2: Frequency distribution of low CR1 expression (*L*) allele in global populations.**

The map shows a significant increase in the frequency of the *L* allele in global populations where *P. vivax* transmission is stable and endemicity of *P. vivax* is shown as colour codes described on the map. The map has been obtained from a previous published research article with permission^2^. *L* allele data was obtained for 34,625 samples from 177 sites in 61 countries on five continents (America, Europe, Asia, Africa, and Australia). Black dots show populations with a significant increase in *L* allele frequency compared with malaria-free areas (*L*≥0.29, p<0.05), whereas blue dots are indicative of a non-significant increase.


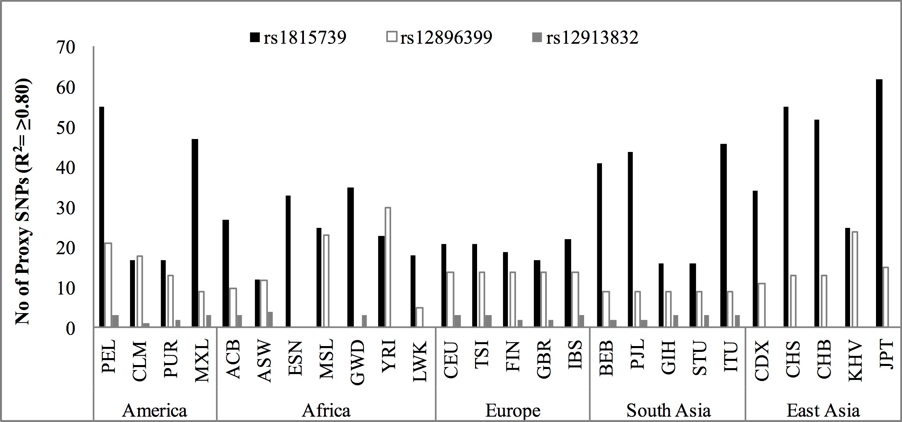


**Figure S3: Proxy SNPs analysis for three SNPs independently associated with human diseases (other than malaria) in global populations shows random numbers of proxy SNPs in world populations.** To confirm the specificity of our results we performed a proxy SNP test of three non-malaria-related SNPs using 1000 Genome Project populations: 1) rs1815739 (*ACTN3* gene, chromosome 11) a premature stop codon in the alpha-actinin-3 gene responsible for muscle endurance ^3^, 2) rs12896399 (SLC24A4 gene, chromosome 14) associated with hair colour ^4^, and 3) rs12913832 (*OCA2* gene, chromosome 15) associated with eye colour ^4^. A 200 Kb region spanning respective SNPs was scanned for R^2^ estimation.

**Table S1:** *Plasmodium vivax* isolates used for *ex vivo* invasion assays.

| Isolates | Parasite density (%) | *ex vivo* invasion assays | | | Parasitemia of control well |
| --- | --- | --- | --- | --- | --- |
|  |  | Enzymatic treatment | sCR1 | Low/High CR1 |  |
| Thai_Plu1037 | 0.2 | √ |  |  | 4.18 |
| Thai_VHX428 | 0.8 | √ |  |  | 4.46 |
| Thai_DMA 38 | 0.2 | √ |  |  | 0.416 |
| Thai_WPP6129 | 0.2 | √ | √ | √ | 9.2 |
| Thai_PID105119 | 0.3 | √ | √ | √ | 1.27 |
| Thai_WPP418 | 0.4 |  | √ | √ | 8.9 |
| Thai_WPP3769 | 0.3 |  | √ | √ | 2.4 |
| Thai_VHX 492 | 0.2 |  | √ | √ | 0.516 |
| Thai_VHX110 | 0.2 |  | √ | √ | 0.466 |
| Thai_WPP188 | 0.7 |  | √ |  | 0.591 |
| Thai_PVITM07 | 0.3 |  | √ |  | 0.479 |
| Peru_Pv08 | 0.9 |  | √ |  | 0.644 |

Abbreviations: sCR1: soluble recombinant CR1 protein. Low/High CR1: Invasion rates of cells expressing high CR1 levels (as measure by flow cytometer) were compared to those expressing low levels.

**Table S2. Linkage disequilibrium between exon 22 (rs2274567) SNP and intron 27 (rs11118133) SNP in global populations.**

| Geographical region | Country | Sample size | D' | R2 |
| --- | --- | --- | --- | --- |
| America | Mexico (MXL) * | 90 | 1.0 | 1.0 |
|  | Puerto Rico (PUR) * | 104 | 0.977 | 0.954 |
|  | Medellin, Colombia (CLM) * | 94 | 1.0 | 0.944 |
|  | Lima, Peru (PEL) * | 85 | 1.0 | 0.969 |
|  | Caucasian_USA ^5^ * | 85 | 1.0 | 0.964 |
|  | African American ^5^ * | 75 | 1.0 | 0.963 |
| Europe | Central Europe (CEU) | 99 | 1.0 | 0.959 |
|  | Toscany, Italy (TSI) | 107 | 1.0 | 0.970 |
|  | Finland (FIN) | 99 | 1.0 | 0.971 |
|  | Britain (GBR) | 91 | 1.0 | 1.0 |
|  | Spain (Iberian pop) (IBS) | 107 | 1.0 | 1.0 |
|  | Sardinia, Italy ^6^ * | 148 | 1.0 | 1.0 |
|  | Italy ^7^ * | 164 | 0.978 | 0.899 |
| Africa | African Ancestry (ASW) | 61 | 1.0 | 1.0 |
|  | Ibadan, Nigeria (YRI) | 108 | 1.0 | 0.976 |
|  | Esan, Nigeria (ESN) | 99 | 1.0 | 0.975 |
|  | Webuye, Kenya (LWK) | 99 | 1.0 | 0.938 |
|  | The Gambia (MAG) | 113 | 0.971 | 0.890 |
|  | Mende, Sierra Leone (MSL) | 85 | 1.0 | 0.887 |
|  | African Caribbean (ACB) | 96 | 0.968 | 0.908 |
| South Asia | Punjab, Pakistan (PJL) | 96 | 1.0 | 0.979 |
|  | Gujarat, India (GIH) | 103 | 1.0 | 1.0 |
|  | Andhra Pradesh, India (ITU) | 102 | 1.0 | 0.981 |
|  | Sri Lanka (STU) | 102 | 1.0 | 0.961 |
|  | Bangladesh (BEB) | 86 | 1.0 | 1.0 |
| East Asia | Japan ^8^ * | 29 | 1.0 | 0.824 |
|  | Japan (JPT) | 104 | 1.0 | 0.970 |
|  | Ho Chi Minh City, Vietnam (KHV) | 99 | 1.0 | 0.979 |
|  | Beijing, China (HAN) (CHB) | 103 | 1.0 | 1.0 |
|  | Southern Han Chinese (CHS) | 105 | 1.0 | 1.0 |
|  | Xishuandbanna, China (CDX) | 93 | 1.0 | 0.978 |

SNP data was obtained from the 1000 Genomes Project Phase 3 database and published articles. Linkage disequilibrium was measured with the Ensembl database tool (<http://www.ensembl.org/>) for biallelic SNPs and with CubeX software for non-biallelic SNPs (*) or SNP data retrieved from published articles.

**Table S3: CR1 L allele frequencies in global populations.**

| Group | Country | City/Ethnic population | Malaria exposure  (Historical/ Current) | Sample size | Malaria parasite | Frequency (L allele) | Locus  (L allele) | References |
| --- | --- | --- | --- | --- | --- | --- | --- | --- |
| No Malaria | UK | Orkney Islands | No/No | 16 | None | 0.13 | Intr26 | 9 |
|  | China | Uyghur | No/No | 10 | None | 0.15 | Intr26 | 9 |
|  |  | Xibe | No/No | 9 | None | 0.22 | Intr26 | 9 |
|  | Russia | Siberia (Yakut) | No/No | 25 | None | 0.20 | Intr26 | 9 |
|  |  | Vologda | No/No | 25 | None | 0.22 | Intr26 | 9 |
| Africa | Barbados | African origin | Yes/No | 96 | Pf | 0.21 | Ex22, Intr27 | 10 |
|  | Tunisia | Tunis**^+^** | Yes/No | 76 | Pf | 0.23 | Ex22 | 11 |
|  | African Origin | Boston^+^ | Yes/No | 75 | Pf | 0.24 | Ex22, Intr27 | 5 |
|  |  | Philadelphia^+^ | Yes/No | 57 | Pf | 0.21 | Intr27 | 12 |
|  | Mali | Bamako^+^ | Yes/Yes | 149 | Pf | 0.15 | Ex22 | 13 |
|  |  | Bandiagara (+3 sites) | Yes/Yes | 515 | Pf | 0.15 | Intr27 | ^14^, ^13^ |
|  | Nigeria | Ibadan | Yes/Yes | 180 | Pf | 0.26 | Ex22, Intr27 | 10 |
|  |  | Esan | Yes/Yes | 99 | Pf | 0.28 | Ex22, Intr27 | 10 |
|  | Kenya | Webuye | Yes/Yes | 100 | Pf | 0.20 | Ex22, Intr27 | 10 |
|  | Gambia | Western Division | Yes/Yes | 113 | Pf | 0.20 | Ex22, Intr27 | 10 |
|  |  | Banjul and Fajara | Yes/Yes | 1276 | Pf | 0.19 | Intr27 | 15 |
|  | Sierra Leone | Mende | Yes/Yes | 85 | Pf | 0.19 | Ex22, Intr27 | 10 |
|  | DR Congo | Ituri | Yes/Yes | 5 | Pf | 0.20 | Intr27 | 9 |
|  | CA Republic | Bordering to R. Congo | Yes/Yes | 5 | Pf | 0.20 | Intr27 | 9 |
|  | Malawi | Karonga**^+^** | Yes/Yes | 177 | Pf | 0.23 | Ex22 | 16 |
|  | Botswana | Sotho-Tswana speaking | Yes/Yes | 22 | Pf | 0.23 | Intr27 | 9 |
|  |  | Nguni ethnic | Yes/Yes | 14 | Pf | 0.28 | Intr27 | 9 |
|  | Namibia | Kalahari Desert | Yes/Yes | 15 | Pf | 0.10 | Intr27 | 9 |
|  | South Africa | Tsonga speaking people | Yes/Yes | 14 | Pf | 0.23 | Intr27 | 9 |
| Europe | European | **Living in USA** | Yes/No | 593 | Pv/Pf | 0.21 | Ex22 | 17 |
|  |  | **Living in USA** | Yes/No | 7607 | Pf/Pv | 0.18 | Intr26 | 18 |
|  | UK | Edinburgh | Yes/No | 60 | Pv | 0.23 | Ex22 | 19 |
|  |  | London**^+^** | Yes/No | 50 | Pv | 0.27 | Intr27 | 20 |
|  |  | **English** | Yes/No | 367 | Pv | 0.15 | Ex22, Intr26 | 6 |
|  | Ireland | **Not defined** | Yes/No | 137 | Pv | 0.15 | Ex22, Intr26 | 6 |
|  |  | Irish | Yes/No | 22 | Pv | 0.16 | Intr26 | 9 |
|  | Norway | Not defined | Yes/No | 102 | Pv | 0.14 | Ex22, Intr26 | 6 |
|  | Finland | Not defined | Yes/No | 99 | Pv | 0.22 | Ex22, Intr27 | 10 |
|  |  | **Oulu^+^** | Yes/No | 98 | Pv | 0.19 | Intr27 | 21 |
|  |  | **Finns** | Yes/No | 25 | Pv | 0.18 | Intr26 | 9 |
|  | Denmark | Danes | Yes/No | 43 | Pv | 0.19 | Intr26 | 9 |
|  | Estonia | Not defined | Yes/No | 976 | Pv | 0.20 | Intr26 | 9 |
|  | France | Not defined**^+^** | Yes/No | 84 | PvPf | 0.21 | Intr27 | 22 |
|  |  | **French** | Yes/No | 29 | PvPf | 0.26 | Intr26 | 9 |
|  |  | **Basque** | Yes/No | 24 | PvPf | 0.10 | Intr26 | 9 |
|  | Belgium | Antwerp | Yes/No | 28 | PvPf | 0.19 | Ex22 | Present study |
|  | Netherland | Nieuwegein**^+^** | Yes/No | 112 | PvPf | 0.24 | Ex22 | 23 |
|  | Czech R | Olomouc**^+^** | YesNo | 203 | Pv/Pf | 0.18 | Ex22 | 23 |
|  | Poland | Lublin^+^ | Yes/No | 852 | PvPf | 0.21 | Ex22, Intr27 | 24 |
|  | Italy | Pavia**^+^** | Yes/No | 900 | PvPf | 0.22 | Ex22 | 25 |
|  |  | **Pavia^+^** | Yes/No | 91 | PvPf | 0.22 | Ex22, Intr27 | 26 |
|  |  | Bologna and Forli**^+^** | Yes/No | 166 | PvPf | 0.18 | Ex22, Intr27 | 7 |
|  |  | **Bergamo** | Yes/No | 13 | PvPf | 0.19 | Intr26 | 9 |
|  |  | **North Italy** | Yes/No | 395 | PvPf | 0.18 | Ex22, Intr26 | 6 |
|  |  | Campania | Yes/No | 8 | PvPf | 0.31* | Intr26 | 9 |
|  |  | South Italy | Yes/No | 220 | PvPf | 0.23 | Ex22, Intr26 | 6 |
|  |  | Sardinia | Yes/No | 148 | PfPv | 0.58*** | Ex22, Intr26 | 6 |
|  |  | Lanusei, Sardinia | Yes/No | 6081 | PfPv | 0.59*** | Ex22 | 27 |
|  |  | Sardinia | Yes/No | 26 | PfPv | 0.64*** | Intr26 | 9 |
|  | Albania | Living in Athens | Yes/No | 12 | PfPv | 0.42*** | Ex22 | Present study |
|  | Greece | Athens | Yes/No | 83 | PfPv | 0.34* | Ex22 | Present study |
|  | Hungary | Debrecen**^+^** | Yes/No | 47 | PvPf | 0.21 | Intr27 | 28 |
|  |  | **Gypsies (Northeast)** | No/No | 41 | PvPf | 0.13 | Intr26 | 9 |
|  | Spain | Iberian (IBS) | Yes/No | 107 | PvPf | 0.17 | Ex22, Intr27 | 10 |
|  | Russia | Krasnodar (Adygei) | Yes/No | 17 | Pv | 0.18 | Intr26 | 9 |
|  |  | Chuvash | No/No | 41 | None | 0.16 | Intr26 | 9 |
|  |  | Tomsk region | Yes/No | 21 | Pv | 0.12 | Intr26 | 9 |
| America | Mexico | Living in USA | Yes/No | 90 | Pv | 0.24 | Ex22, Intr27 | 10 |
|  |  | Sonora | Yes/No | 25 | Pv | 0.26 | Intr26 | 9 |
|  |  | Mexico City**^+^** | Yes/No | 11 | Pv | 0.32* | Intr27 | 29 |
|  |  | Yutican | Yes/Yes | 24 | Pv | 0.52*** | Intr26 | 9 |
|  | Colombia | Medellin | No/No | 94 | None | 0.27 | Ex22, Intr27 | 10 |
|  |  | Choco | Yes/Yes | 25 | Pv | 0.44*** | Ex22 | Present study |
|  |  | Cordoba | Yes/Yes | 29 | Pv | 0.34* | Ex22 | Present study |
|  |  | Amerindian | Yes/Yes | 13 | Pv | 0.38** | Intr26 | 9 |
|  | Peru | Lima | Yes/No | 85 | Pv | 0.27 | Ex22, Intr27 | 10 |
|  |  | Iquitos | Yes/Yes | 76 | Pv | 0.37** | Ex22 | Present study |
|  |  | Andoas | Yes/Yes | 77 | Pv | 0.31* | Ex22 | Present study |
|  |  | San Jose | Yes/Yes | 16 | Pv | 0.44*** | Ex22 | Present study |
|  | Puerto Rico | Puerto Ricon | Yes/No | 104 | Pv | 0.30* | Ex22, Intr27 | 10 |
|  | Brazil | Randonia (Karitiana) | Yes/Yes | 25 | Pv | 0.56*** | Intr26 | 9 |
|  |  | Randonia (Surui) | Yes/Yes | 21 | Pv | 0.50*** | Intr26 | 9 |
|  |  | Amazon, Ticuna | Yes/Yes | 34 | Pv | 0.21 | Intr26 | 9 |
|  |  | Ceara (Living in Acre) | No/Yes | 100 | Pv | 0.26 | Ex22 | Present study |
|  | Bolivia | Quechua | Yes/No | 23 | Pv | 0.26 | Intr26 | 9 |
| West Asia | Israel | Jews (Living Europe) | Yes/No | 120 | Pv/Pf | 0.28 | Ex22, Intr26 | 6 |
|  |  | Negev | Yes/No | 48 | PvPf | 0.29* | Intr26 | 9 |
|  |  | Palestinian | Yes/No | 51 | PvPf | 0.27 | Intr26 | 9 |
|  |  | Samaritan | Yes/No | 38 | Pv/Pf | 0.47*** | Intr26 | 9 |
|  |  | Jews (Yemen) | Yes/No | 37 | Pv/Pf | 0.35** | Intr26 | 9 |
|  |  | Jews (Germany) | Yes/No | 24 | Pv/Pf | 0.25 | Intr26 | 9 |
|  |  | Jews (Ethiopia) | Yes/No | 31 | Pv/Pf | 0.21 | Intr26 | 9 |
|  | Syria/Israel | Northern | Yes/No | 47 | PvPf | 0.19 | Intr26 | 9 |
|  | Arab | Not defined | Yes/No | 113 | None | 0.16 | Ex22, Intr26 | 6 |
| East Asia | Russia | Khanty | No/No | 47 | None | 0.13 | Intr26 | 9 |
|  |  | Komi-Zyrian | No/No | 46 | None | 0.14 | Intr26 | 9 |
|  | Japan | Japanese | Yes/No | 104 | Pv | 0.19 | Ex22, Intr27 | 10 |
|  |  | Tokyo | Yes/No | 751 | Pv | 0.20 | Ex22 | 30 |
|  |  | **Japanese (CHMJ)** | Yes/No | 37 | Pv | 0.20 | Ex22 | 31 |
|  |  | **Tokyo^+^** | Yes/No | 28 | Pv | 0.23 | Intr26 | 9 |
|  |  | Kyushu, Fukuoka**^+^** | Yes/No | 52 | Pv | 0.20 | Intr27 | 32 |
|  |  | **Japanese** | Yes/No | 19 | Pv | 0.15 | Intr27 | 9 |
|  | Korea | Seoul | YesNo | 53 | Pv | 0.14 | Intr26 | 9 |
|  | Taiwan | Living in USA | Yes/No | 93 | Pv | 0.29* | Intr27 | 14 |
|  |  | Ami | Yes/No | 38 | Pv | 0.44*** | Intr26 | 9 |
|  |  | Hakka | Yes/No | 37 | Pv | 0.38** | Intr26 | 9 |
|  |  | Atayal | Yes/No | 34 | Pv | 0.162 | Intr26 | 9 |
|  | China | **Beijing^+^** | Yes/No | 212 | Pv | 0.18 | Intr27 | 33 |
|  |  | Beijing (Han) | Yes/No | 103 | Pv | 0.17 | Ex22, Intr27 | 10 |
|  |  | Southern Han (CHB) | Yes/No | 115 | Pv | 0.33* | Ex22, Intr27 | 10 |
|  |  | Xishuangbana | Yes/No | 93 | Pv | 0.59*** | Ex22, Intr27 | 10 |
|  |  | Henan**^+^** | Yes/No | 60 | Pv | 0.61*** | Ex22 | 34 |
|  |  | Guangxi**^+^** | Yes/No | 1012 | Pv | 0.47*** | Ex22 | 35 |
|  |  | Living in Singapore | Yes/No | 96 | Pv | 0.31* | Intr26 | 36 |
|  |  | Dai | Yes/No | 10 | Pv | 0.55*** | Intr26 | 9 |
|  |  | Daur | Yes/No | 9 | Pv | 0.28 | Intr26 | 9 |
|  |  | **Han** | Yes/No | 43 | Pv | 0.20 | Intr26 | 9 |
|  |  | **Han** | Yes/No | 17 | Pv | 0.21 | Intr27 | 9 |
|  |  | Hezhe | Yes/No | 9 | Pv | 0.17 | Intr26 | 9 |
|  |  | Lahu | Yes/No | 10 | Pv | 0.65*** | Intr26 | 9 |
|  |  | Miao | Yes/No | 10 | Pv | 0.30* | Intr26 | 9 |
|  |  | Naxi | Yes/No | 9 | Pv | 0.17 | Intr26 | 9 |
|  |  | Oroqen | Yes/No | 10 | Pv | 0.35** | Intr26 | 9 |
|  |  | She | Yes/No | 10 | Pv | 0.30* | Intr26 | 9 |
|  |  | Tu | Yes/No | 10 | Pv | 0.25 | Intr26 | 9 |
|  |  | Tujia | Yes/No | 10 | Yes | 0.15 | Intr26 | 9 |
|  |  | Yi | Yes/No | 10 | Pv | 0.30* | Intr26 | 9 |
|  | Mongolia | **Northeast** | No/No | 11 | None | 0.05 | Intr26 | 9 |
| South Asia | Pakistan | Lahore | Yes/Yes | 96 | Pv | 0.45*** | Ex22, Intr27 | 10 |
|  |  | Burusho | Yes/No | 25 | Pv | 0.24 | Intr26 | 9 |
|  |  | Kalash | Yes/No | 25 | Pv | 0.26 | Intr26 | 9 |
|  |  | Brahui | Yes/Yes | 25 | Pv | 0.46*** | Intr26 | 9 |
|  |  | Sindh | Yes/Yes | 25 | Pv | 0.30* | Intr26 | 9 |
|  | Pak/Afghan | Pashtun | Yes/Yes | 23 | Pv | 0.39** | Intr26 | 9 |
|  |  | Hazara | Yes/Yes | 24 | Pv | 0.25 | Intr26 | 9 |
|  | Pak/Iran | **Baloch** | Yes/Yes | 25 | Pv | 0.36** | Intr26 | 9 |
|  |  | Baloch | Yes/Yes | 25 | Pv | 0.42** | Intr26 | 9 |
|  | India | Gujarati in USA | Yes/No | 100 | Pv | 0.49*** | Ex22, Intr27 | 10 |
|  |  | Ratnagiri | Yes/Yes | 2042 | Pv/Pf | 0.38** | Ex22 | 37 |
|  |  | Cuttack^H^ | Yes/Yes | 100 | PfPv | 0.53*** | Ex22, Intr27 | 38 |
|  |  | **Cuttack$** | Yes/Yes | 210 | PfPv | 0.54*** | Ex22, Intr27 | 39 |
|  |  | Delhi**^+^** | Yes/Yes | 218 | PfPv | 0.49*** | Intr27 | ^40^, ^41^ |
|  |  | Antagarh and Sundargarh^H^ | Yes/Yes | 102 | PfPv | 0.59*** | Ex22, Intr27 | 42 |
|  |  | **Lucknow^H^** | Yes/Yes | 84 | PfPv | 0.39** | Ex22, Intr27 | 42 |
|  |  | Lucknow**^+^** | Yes/Yes | 200 | PfPv | 0.43*** | Ex22, Intr27 | 43 |
|  |  | Andhra Pradesh | Yes/No | 102 | PfPv | 0.56*** | Ex22, Intr27 | 10 |
|  |  | Chennai**^+^** | Yes/Yes | 125 | Pv | 0.44*** | Intr27 | 44 |
|  |  | Living in Singapore | Yes/No | 83 | Pv | 0.48*** | Intr26 | 36 |
|  | Bangladesh | Bengali | Yes/Yes | 86 | PfPv | 0.44*** | Ex22, Intr27 | 10 |
|  | Sri Lanka | Living in USA | Yes/No | 102 | Pv | 0.53*** | Ex22, Intr27 | 10 |
| South E. Asia | Thailand | Northwest Thailand | Yes/Yes | 477 | PfPv | 0.64*** | Ex22, Intr27 | 45 |
|  |  | Bangkok | Yes/Yes | 215 | PfPv | 0.46*** | Ex22 | 46 |
|  |  | Maesot | Yes/Yes | 27 | PfPv | 0.43*** | Ex22 | Present study |
|  | Cambodia | Mondulkiri | Yes/Yes | 49 | PfPv | 0.53*** | Intr27 | 14 |
|  |  | Phnom Penh | Yes/Yes | 135 | PfPv | 0.50*** | Intr27 | 14 |
|  |  | Sam PeovLoum | Yes/Yes | 79 | PfPv | 0.46*** | Intr27 | 14 |
|  |  | Ratanakiri | Yes/Yes | 41 | PfPv | 0.42*** | Intr27 | 14 |
|  |  | **Khmer (Living USA)** | Yes/Yes | 15 | PfPv | 0.36** | Intr26 | 9 |
|  |  | **Khmer (Living USA)** | Yes/Yes | 12 | PfPv | 0.33* | Intr27 | 9 |
|  | Vietnam | Ho Chi Minh City | Yes/Yes | 99 | PfPv | 0.38** | Ex22, Intr27 | 10 |
|  |  | Quang Nom | Yes/Yes | 95 | PfPv | 0.42*** | Ex22 | Present study |
|  |  | Living in USA | Yes/Yes | 9 | PfPv | 0.44*** | Intr27 | 9 |
|  | Laos | Lao Loum | Yes/Yes | 59 | PfPv | 0.45*** | Intr26 | 9 |
|  | Malaysia | MalayAborigine | Yes/Yes | 6 | PfPvPk | 0.58*** | Intr27 | 9 |
|  |  | Living in Singapore | Yes/Yes | 89 | PfPvPk | 0.40*** | Intr26 | 36 |
|  | Indonesia | Jakarta**^+^** | Yes/Yes | 320 | PfPv | 0.44*** | Ex22 | 47 |
| Asia Pacific | PNG | New Ireland Province | Yes/Yes | 47 | PfPv | 0.73*** | Ex22 | 19 |
|  |  | Madang, Madang Province (MP) | Yes/Yes | 69 | PfPv | 0.58*** | Intr27 | 14 |
|  |  | **Madang, (MP)** | Yes/Yes | 358 | PfPv | 0.72*** | Ex22 | 19 |
|  |  | Mugil/Megiar, (MP) | Yes/Yes | 206 | PfPv | 0.65*** | Ex22 | 48 |
|  |  | Eastern Highland Province | Yes/Yes | 17 | PfPv | 0.41*** | Ex22 | 19 |
|  |  | Amele, (MP) | Yes/Yes | 530 | PfPv | 0.77*** | Ex22 | 49 |
|  |  | Coastal region | Yes/Yes | 17 | PfPv | 0.59*** | Intr26 | 9 |
|  |  | Bougainville | Yes/Yes | 19 | PfPv | 0.76*** | Intr26 | 9 |
|  | Micronesia | Micronesians | Yes/No | 33 | PfPv | 0.26 | Intro26 | 9 |
|  | Australia | Not described | Yes/No | 80 | PfPv | 0.17 | Ex22 | 50 |
| **Total** | **61 countries** | 177 different sites |  | 34625 |  |  |  |  |

**Description:**

Country column: location of a population on the geographical map was determined based on human ethnicity. For example, a person of European origin living in USA was added in Europe. City/region column, **^+^:** indicate CR1 data from healthy control subjects recruited to study Systemic lupus erythematosus (SLE), Tuberculosis (TB) and other human diseases, H: healthy control from malaria association studies, $: samples from mild malaria cases (no healthy control), bold name: population excluded in figure 5. Malaria exposure column: historical exposure refers to populations whose ancestors were exposed to malaria (before 1945 when WHO launched the malaria eradication program) but are currently free malaria regions. Current exposure indicates recent exposure to Plasmodium species. Both historical and current malaria exposure data was extracted from previously published data ^2,51-54^. Malaria parasite column: indicates transmission of human malaria species which was determined based on previous publications ^2,54^. Pf: *P. falciparum*, Pv: *P. vivax* and Pk: *P. knowlesi.* *: P values (*: <0.05, **: <0.005 and ***: <0.0001) was obtained by comparing mean *L* allele frequency of malaria-free populations (L=0.17) with all populations. Malaria free populations in this study are: Vologda, Siberia, Orkney Islands, Xibe and Uyghur. Locus column indicate SNPs that were used to determine *L* allele.

In order to remove selection bias due to associations between *L* alleles and other human diseases, only CR1 genotype data from healthy control individuals or individuals with disease but with allele frequencies that were non-significantly different from those in healthy controls (p <0.05%) were included in the allele frequency analysis.

**Table S4: Complement receptor 1 PCR primers, amplification conditions and RFLP protocol.**

| **SNP** | **Primers** | **Sequences (5’ - 3’)** | **PCR product**  **(bp)** | **PCR-RFLP**  ***Rsa*1 products (bp)** | |
| --- | --- | --- | --- | --- | --- |
| Rs2274567  (gDNA) | Xiang et al ^5^ | Xiang et al ^5^ | 700 | 520+180 (HH), 520+480+180 (HL), 480+180 (LL) | |
|  |  |  |  |  |  |
| Rs2274567 | CR1ex22-F | tgtgtcagccgcctccag | 177 | 139+38 (HH), 177 (LL), 177 +139+38 (HL) | |
| (cDNA) | CR1ex22-R | ctgcacatctcggggctt |  |  |  |
| Rs11118131 | Intr26-F | gtaggtgggatttgattc | 291 | 151+91+47 (HH), 244+47 (LL), 244+151+91+47 (HL) | |
| (intron 26) | Intr26-R | ttagttagccaagcacag |  |  |  |
| PCR Thermo profile | | | | | |
| Stage | Step | Temperature | | | Time |
| Holding | Initial denaturation | 95°C | | | 3 min |
| Cycling (35x) | Denaturation | 95°C | | | 20 sec |
|  | Annealing | 48°C (Exon 22: cDNA) or 50ºC (in26) | | | 30 sec |
|  | Extension | 72°C | | | 30 sec |
| Holding | Final extension | 72°C | | | 5 min |

CR1 exon 22 genotyping from gDNA was done as reported earlier ^5^. The additional CR1ex22 primers were used for amplifying exon 22 from cDNA. HH: wild type homozygote (high CR1 expression), LL: mutant type homozygote (low CR1 expression), HL: Heterozygote (intermediate or low to high CR1 expression).

**References:**
